# Supplementary material for: Co-reviewing and ghostwriting by early-career researchers in the peer review of manuscripts
Source: eLife. 2019 Oct 31;8:e48425. doi: 10.7554/eLife.48425 (PMC6822987; doi:10.7554/eLife.48425)
Supplement: Supplementary file 1. [file elife-48425-supp1.docx]

**SUPPLEMENTARY FILE 1**

**Results of relevance screening for literature review**

List of all search records categorized by initial screeners as "relevant" and/or "maybe relevant" to the topic of ECR involvement in the peer review of manuscripts based on title and abstract (n = 118). Y = yes, relevant; M = maybe relevant; N = not relevant; - = not evaluated.

| **Article title**  (alphabetical order) | **Initial Screeners** | | **Tie- breaker** | **Full Text Reading** |
| --- | --- | --- | --- | --- |
|  | **#1** | **#2** | **#3** |  |
| A Fine Balance: How Authors Strategize Around Journal Submission | N | M | - |  |
| A guide to critiquing a research paper. Methodological appraisal of a paper on nurses in abortion care | M | N | - |  |
| A Mentoring Opportunity: A Joint Effort in Writing Letters of Recommendation | N | Y | N |  |
| A model for scholarship in nursing: The case of a private liberal arts college | N | M | - |  |
| A Pragmatic Approach to Getting Published: 35 Tips for Early Career Researchers | M | Y | Y | Y |
| A Survey of Methods for Improving Review Quality | N | M | - |  |
| A web-based synchronous collaborative review tool: A case study of an on-line graduate course | N | M | - |  |
| Advise to research trainees in biomedical sciences - A very personal tribute to the late Dr. Susumu Ohno | N | Y | N |  |
| An ounce of prevention: An associate editor's view | N | M | - |  |
| Assessing postgraduate student perceptions and measures of learning in a peer review feedback process | M | M | N |  |
| Assessment for Learning Research and Writing Skills through Scaffolded Online Peer Review | N | M | - |  |
| Becoming a peer reviewer: engaging in sharing and gaining knowledge | M | Y | Y | Y |
| Being a researcher is not only a matter of publishing: Learning to review scientific articles | Y | Y | Y | Y |
| Biosecurity and the review and publication of dual-use research of concern | N | M | - |  |
| Building Scholarly Writers: Student Perspectives on Peer Review in a Doctoral Writing Seminar | M | M | N |  |
| Calibrated Peer Review: Enabling Technology for a Large - Scale Writing Intensive Neuroscience Course. | M | M | N |  |
| Calibrated Peer Review: Implementation of a computer-based writing-to-learn tool in a large lecture-based introductory biology class | M | M | N |  |
| Calibrated peer review: The application of statistical concepts to a biological research question | N | M | - |  |
| Can Online Peer Review Assignments Replace Essays in Third Year University Courses? And if so, What are the Challenges? | N | M | - |  |
| CAREERS eLife and early career researchers | N | Y | Y | Y |
| Celebrating Peer Review Week | M | M | Y | Y |
| Chinese Early-Career Researchers' Scholarly Communication Attitudes and Behaviours: Changes Observed in Year Two of a Longitudinal Study | M | Y | Y | Y |
| Conducting mental health research: Key steps, practicalities, and issues for the early career researcher | M | N | - |  |
| Developing Knowledge Generation, Communication and Management In Teacher Education: A Successful Attempt of Teaching Novice Computer Users | N | M | - |  |
| Developing laboratory skills by incorporating peer-review and digital badges | N | M | - |  |
| Do peer review models affect clinicians' trust in journals? A survey of junior doctors | M | Y | Y | Y |
| Does mentoring new peer reviewers improve review quality? A randomized trial | Y | Y | Y | Y |
| Does peer review predict the performance of research projects in health sciences? | N | M | - |  |
| Doing peer review and receiving feedback: impact on scientific literacy and writing skills | M | M | Y | Y |
| Doing Peer Review: Reflections From an International Group of Postdoctoral Fellows | M | Y | Y | Y |
| Editorial training models for early-career psychiatrists | N | Y | Y | Y |
| Emergent areas to visualize by the journal strategy holders | M | N | - |  |
| Enhancement of Resident Competencies Via Participation in the Peer Review/Quality Improvement Processes and Morbidity and Mortality Presentations | M | N | - |  |
| Evaluation of multi- and interdisciplinary research - the no-peer problem | N | M | - |  |
| Examining the Effects of Trained Peer Feedback on EFL Students’ Writing | N | M | - |  |
| Expanding Group Peer Review: A Proposal for Medical Education Scholarship | M | Y | Y | Y |
| Facilitating improvements in laboratory report writing skills with less grading: a laboratory report peer-review process | N | M | - |  |
| Feedback on Peer Feedback in EFL Composing: Four Stories | N | Y | N |  |
| Graduate student-run course framework for comprehensive professional development | N | M | - |  |
| Guidelines for assessment of publications for contribution to scholarship: a view point. | N | Y | Y | Y |
| Heroes of peer review: Robert Lowe | N | M | - |  |
| How early-career researchers are shaping eLife | N | Y | Y | Y |
| How to achieve accurate peer assessment for high value written assignments in a senior undergraduate course | N | M | - |  |
| Improving peer reviewing in scientific conferences | M | M | Y | Y |
| Increasing the Quality of Peer Feedback in a Professional Writing Course | N | M | - |  |
| Instructors' uses, experiences, thoughts and suggestions regarding Calibrated Peer Review | M | M | N |  |
| Integrating critical analysis, peer review, and independent research in the laboratory phase of a microbiology course | M | M | N |  |
| Investigating peer review as a systemic pedagogy for developing the design knowledge, skills, and dispositions of novice instructional design students | M | N | - |  |
| Judging plagiarism: a problem of morality and convention | N | Y | Y | Y |
| Korean University Students‚Äô Attitudes towards Peer Review in EFL Writing | N | M | - |  |
| Learning by reviewing | N | Y | Y | Y |
| Literature search in medical publications | N | M | - |  |
| Manuscript peer review: A helpful checklist for students and novice referees. | M | M | Y | Y |
| Mentor/mentee relationship with the focus on meeting promotion/tenure guidelines | N | M | - |  |
| Misrepresentation and responsibility in medical research | M | M | N |  |
| Mixed Method Study Examines Undergraduate Student Researchers’ Knowledge and Perceptions About Scholarly Communication Practices | M | Y | Y | Y |
| Moving from trainee to junior: faculty: a brief guide | M | M | Y | Y |
| Nature neuroscience | N | M | - |  |
| New Reviewer Mentoring Program | N | Y | Y | Y |
| Observations: The Introduction of "Junior Editor" Posts Within Journals | N | M | - |  |
| Open Access Open Grad Students | N | Y | Y | Y |
| Open peer review: a randomised controlled trial | M | M | Y | Y |
| Peer Assessments Using the Moodle Workshop Tool | N | M | - |  |
| Peer review as an educational strategy to improve academic work: An interdisciplinary collaboration between communication disorders and nursing | N | M | - |  |
| Peer Review in Radiology: A Resident and Fellow Perspective | M | N | - |  |
| Peer review process: Guidelines for clinical and forensic psychological reviews | N | M | - |  |
| Peer review: why we need nurse leaders to serve as reviewers for nursing publications | M | M | - |  |
| Peer-Review - using a Paired-Comparison Technique | N | M | - |  |
| Perception of Academic Writing: A Case Study of an ESL Writer during Peer Review | N | M | - |  |
| Perspectives from early career researchers on the publication process in ecology - a response to Statzner & Resh (2010) | M | Y | Y | Y |
| Pit-bull reviewing, the pursuit of perfection and the victims of success | N | M | - |  |
| Plagiarism: an egregious form of misconduct | M | Y | Y | Y |
| Plagiarism. A fools' errand | M | Y | Y | Y |
| Potential of information technology in dental education | N | M | - |  |
| Power of peer review: An online collaborative learning assignment in social psychology | N | M | - |  |
| Preparing the senior or graduating student for graduate research | M | Y | Y | Y |
| PRO myths vs. facts | N | M | - |  |
| Professional Skills Courses Increase Trainee Skills in Writing, Reviewing, Networking, and Poster Presentations | M | M | N |  |
| Professionalism and Communication Education in Pediatric Critical Care Medicine: The Learner Perspective | M | N | - |  |
| Promoting operational research through fellowships: a case study from the South-East Asia Union Office | M | N | - |  |
| Publish or Perish: A Mandate With Negative Collateral Consequences | M | N | - |  |
| Pursuing the journal mission | M | Y | Y | Y |
| Religion, Rebel Scientists, and Peer-Review - 3 Hot Topics | N | M | - |  |
| Replacing the Traditional Graduate Chemistry Literature Seminar with a Chemical Research Literacy Course | M | N | - |  |
| Republished paper: Assuring validity of multisource feedback in a national programme | M | N | - |  |
| Research as a subject--for research | N | M | - |  |
| Research Ethics II: Mentoring, Collaboration, Peer Review, and Data Management and Ownership | N | Y | Y | Y |
| Responsible Conduct of Research in Communication Sciences and Disorders: Faculty and Student Perceptions | M | M | Y | Y |
| Review, revise, and resubmit: The effects of self-critique, peer review, and instructor feedback on student writing | N | M | - |  |
| Reviewing a Manuscript: Disparity Amongst Peer Reviewers' Priorities from Basic Health Sciences and Clinicians | M | Y | Y | Y |
| Student peer review decisions on submitted manuscripts are as stringent as faculty peer reviewers | Y | Y | Y | Y |
| Supersizing e-learning: What a CoI survey reveals about teaching presence in a large online class | N | M | - |  |
| Survey of publication outlets in early childhood education: Descriptive data, review processes, and advice to authors | M | N | - |  |
| The "Peer" in "Peer Review" | N | M | - |  |
| The 5th Annual AABT Postdoctoral Panel and Overview: November 2004 | N | M | - |  |
| The Acta Psychiatrica Scandinavica Trainee Advisory Board: Education, mentoring, and experience with the editorial process | M | Y | Y | Y |
| The association between four citation metrics and peer rankings of research influence of Australian researchers in six fields of public health | M | M | N |  |
| The ethics of peer review: What to know before saying "yes" | M | Y | Y | Y |
| The Fox and the Crow' or 'the Foolishness of Vanity Publishing in Fake Academic Journals': A Story from the Arabian Gulf | N | M | - |  |
| The impact of E-learning in medical education | N | M | - |  |
| The invited review ? or, my field, from my standpoint, written by me using only my data and my ideas, and citing only my publications | M | N | - |  |
| The lecturer should know what they are talking about: Student union officers perceptions of teaching-related CPD and implications for their practice | N | M | - |  |
| The More You Know: The Impact of Publication and Peer-Review Experience on Psychology Graduate Students | M | Y | Y | Y |
| The peer review gap: A longitudinal case study of gendered publishing and occupational patterns in a female-rich discipline, Western North America (1974-2016) | M | Y | N |  |
| THE PIPELINE Scientific Teaching in Practice | N | M | - |  |
| The planning and implementation of a faculty peer review teaching project | N | M | - |  |
| The Use of Mock NSF-type Grant Proposals and Blind Peer Review as the Capstone Assignment in Upper-Level Neurobiology and Cell Biology Courses. | M | M | N |  |
| The value of peer feedback opportunities for students in writing intensive classes | N | Y | N |  |
| Three tough acts to follow | N | M | - |  |
| Two Modes of Peer Review among Adult Writers: Which Is Better? | N | M | - |  |
| Use of Peer-Review System for Enhancing Learning of Programming | N | M | - |  |
| Using an Undergraduate Immunology Laboratory Course to Integrate Scholarship and Teaching | N | M | - |  |
| Using Calibrated Peer Review to Teach Basic Research Skills | M | M | N |  |
| Validity and reliability of scaffolded peer assessment of writing from instructor and student perspectives | N | M | - |  |
| Variability in students' evaluating processes in peer assessment with calibrated peer review | M | Y | N |  |
| Where does all the research go? Reflections on supporting trainee-applied psychologists to publish their research | M | Y | N |  |
| Why not assess another trainer and his/her trainee? | N | M | - |  |
| Writing to learn: an evaluation of the calibrated peer review program in two neuroscience courses | M | Y | Y | Y |
