# Supplementary material for: Co-reviewing and ghostwriting by early-career researchers in the peer review of manuscripts
Source: eLife. 2019 Oct 31;8:e48425. doi: 10.7554/eLife.48425 (PMC6822987; doi:10.7554/eLife.48425)
Supplement: Supplementary file 2. [file elife-48425-supp2.docx]

**SUPPLEMENTARY FILE 2**

**Text of The Role of Early Career Researchers in Peer Review - Survey**

**Background**

Peer review of academic manuscripts is essential to maintain integrity in science and is integral to the journal publication process. Early Career Researchers (ECRs) often contribute to this peer review process. While ECRs may review manuscripts jointly with or under the direction of a senior academic, such as a Principal Investigator (PI), Group Leader, or Professor, a large number of ECRs claimed in a recent survey to have acted as peer review “ghostwriters”; that is, the peer review report (i.e. the final review submitted to the journal editor) had only the senior academic’s name attributed to the report. For the rest of this survey, we refer to the senior academic as the “PI” and any junior academics under their supervision as “ECRs.”

This survey is designed to collect more data about the phenomenon of ghostwriting by ECRs. The goal of this survey is to assess the experiences and opinions of the community, and to recommend best practices for recognizing co-reviewing activities.

This survey contains 16 questions and is estimated to take 15 minutes.

**Statements of Disclosure, Ethics and Informed Consent**

This survey was created by researchers affiliated with the Future of Research, a non-profit organization in the United States that is promoting an effort to increase transparency about co-reviewing activities by ECRs. You can find out more about our work on ECRs in peer review at our website here: http://futureofresearch.org/ecrpeerreview/

The researchers respect the confidentiality and anonymity of all respondents. No identifiable private information will be collected by this survey. Your participation is voluntary and you can choose to stop at any time. Please complete this survey only once. By choosing to submit answers to this survey, you thereby provide your informed consent to voluntarily share your experiences and opinions with the researchers, who intend to publish a summary of the results of the survey but not the raw data with participants' individual demographic information.

You may contact Gary McDowell, Executive Director of the Future of Research, at futureofresearch@gmail.com at any time during the study if you have questions or concerns about your participation.

This survey has been verified by the Mount Holyoke Institutional Review Board as Exempt according to 45CFR46.101(b)(2): Anonymous Surveys - No Risk on 08/21/2018.

I provide my informed consent to participate in this survey.

- Yes

**Professional Information**

Q1.What is your current institution? Fill in blank box (e.g. Harvard School of Medicine).

Q2. What is your current field of research? Fill in blank box (e.g. biomedicine; physics; philosophy; economics;

etc.).

Q3. What is your current career stage?

- Undergraduate Student
- Graduate Student - Masters
- Graduate Student - PhD
- Postdoctoral Researcher
- Staff Scientist
- Adjunct Professor
- Principal Investigator (PI)
- Other (please describe)

**Demographic Information**

Please feel free to skip any of the following questions if you feel they would be sufficient to uniquely identify you.

Q4.What is your gender identity?

- Female
- Male
- Prefer not to say
- Other (please describe)

Q5. What is your race/ethnicity? Select all that apply.

- Asian
- Black or African American
- Hispanic or Latinx
- Native American or Alaska Native
- Native Hawaiian or Pacific Islander
- White
- Prefer not to say
- Other (please describe)

Q6. If you are based in the U.S., are you a U.S. Citizen/Permanent Resident?

- Yes
- No
- Not based in the U.S

**Your peer review experience**

Q7.How many times in your career have you reviewed an article for publication independently, i.e. carried out the full review and been identified to the editorial staff as the sole reviewer?

- 0
- 1-5
- 6-20
- 21+

Q8.How many times in your career have you contributed ideas and/or text to peer review reports where you are not the invited reviewer (e.g. the invited reviewer is the PI for whom you work)?

- 0 - skip to question 13
- 1-5 - go to question 9
- 6-20 - go to question 9
- 21+ - go to question 9

Q9. When you were not the invited reviewer, what was the extent of your involvement in the peer review process? Please select all that apply to your entire peer review experience (e.g. across multiple manuscripts).

- I read the manuscript, shared short comments with my PI, and was no longer involved
- I read the manuscript, wrote a full report for my PI, and was no longer involved
- I read the manuscript, wrote the report, my PI edited the report and we submitted the report together with both of our names provided to the editorial staff
- I read the manuscript, wrote the report, my PI edited the report and my PI submitted report with only their name provided to the editorial staff
- I read the manuscript, wrote the report, and submitted it independently without my PI’s name provided to the editorial staff

Q10. To your knowledge, did your PI ever submit your reviews without editing your work?

- Yes
- No
- Don’t know

Q11. To your knowledge, did your PI ever withhold your name from the editorial staff when you served as the reviewer or coreviewer?

- Yes - proceed to question 12
- No - skip to question 13
- Don’t know - proceed to question 12

Q12. Consider cases where you contributed to a peer review report and you know your name was NOT provided to the editorial staff. When discussing this with your PI, what reason did they give to exclude you as a co-reviewer?

- Did not discuss with my PI
- Journal does not allow ECRs to review
- Journal requires prior approval to share manuscript, which was not obtained
- Intellectual contribution not deemed sufficient
- Co-authorship is for papers, not for peer review reports
- Other (please describe)

Q13. How did you gain training in how to peer review a manuscript? Select all that apply.

- Online resource
- Your PI
- A postdoc in the lab
- A graduate student in the lab
- Journal Club
- Attending an in-person course/workshop
- From receiving reviews on my own papers
- I have had no training
- Other (please describe)

**Your opinions on peer review**

The following questions are about your opinions, not necessarily your experiences. Please answer the following questions regardless of whether or not you have participated in peer review.

A ghostwriter is defined as a person that writes text or other scholarly works without receiving authorship.

These questions are about submitting names of co-authors to the editorial office, not making the identities of reviewers publicly available.

Q14. Please indicate how strongly you agree with the following statements. You may also submit comments to expand and/or clarify your opinions in the textbox below.

*Options: Strongly Disagree; Slightly Disagree; No Opinion; Slightly Agree; Strongly Agree*

- Involving members of a research group in peer review is a beneficial training exercise.
- It is ethical for the invited reviewer (e.g. PI) to involve others (e.g. their trainees) in reviewing manuscripts.
- It is ethical for the invited reviewer (e.g. PI) to submit a peer review report to an editor without providing the names of all individuals who have contributed ideas and/or text to the report.
- Ghostwriting a peer-review report for your PI is an ethically sound scientific practice.
- When a journal invites a PI to review, that is equivalent to the journal inviting anyone in that PI's research group with relevant expertise to contribute to the review.
- It would be valuable to have my name added to a peer review report (e.g. to be recognized as a co-reviewer by the editor; or to use a service such as Publons to be assigned credit).
- Anyone that contributes ideas and/or text to the review report should be included as a co-author on the review.
- The only person who should be named on a peer review report is the invited reviewer, regardless of who carried out the review.
- Adding names of other contributors to a peer review report diminishes the credibility of the report.
- The current system used to name and order co-authors on manuscripts in my field should also be used to the name and order co-reviewers on peer review reports.
- The current system used to identify author contributions on manuscripts in my field (e.g. AB did the experiments, AB and CD analyzed the data and wrote the paper), or the CRediT taxonomy (https://casrai.org/credit/), should also be used to identify author contributions on peer review reports (e.g. AB reviewed the experiments, AB and CD wrote the report).

Please submit any extra thoughts or comments regarding question 14 here:

Q15. What do you think are the reasons why the names of co-authors on peer review reports  may not be provided to the editorial staff? Please select all that you think apply.

- A lack of a mechanism (such as a textbox, with language demonstrating expectations that co-reviewers be listed) to include this information in the peer review report submission process.
- A belief that reviews should only be done by the invited reviewer, and not by, or with assistance from, anyone else.
- A belief that only the invited reviewer deserves authorship, even when others contribute ideas and/or text to the review report.
- A belief that there is no strong ethical reason to add co-authors names.
- A belief that including co-author information would demonstrate that the PI breached the confidentiality of the manuscript.
- Some ECRs may not be comfortable asking for co-authorship.
- A belief that keeping ECR names off of peer review protects ECRs during a vulnerable time in their career.
- Some co-reviewers want to be able to write critical reviews anonymously.
- A belief that ghostwriting does not occur: everyone always provides the names of all contributing authors to the editorial office.
- A belief that ghostwriting does not occur: PIs are the only people that contribute to peer review reports.
- Other (please describe)

Q16. Would any of the opinions you have just expressed change if the content of peer review reports (i.e. the text of reviews) were published openly alongside the papers? And should such published reports include or exclude the reviewer's name(s)? Please explain.

Thank you!

You have completed the survey. Many thanks for your responses! Please check http://futureofresearch.org/ecrpeerreview/ or subscribe to our blog to keep updated on the results. Please share the link to the survey with your colleagues: <https://tinyurl.com/ECRs-in-peer-review>
